# Revealing the intratumoral heterogeneity of non-DS acute megakaryoblastic leukemia in single-cell resolution

**DOI:** 10.3389/fonc.2022.915833

**Published:** 2022-08-08

**Authors:** Narun Su, Zifeng Li, Jiapeng Yang, Yang Fu, Xiaohua Zhu, Hui Miao, Yi Yu, Wenjin Jiang, Jun Le, Xiaowen Qian, Hongsheng Wang, Maoxiang Qian, Xiaowen Zhai

**Affiliations:** ^1^ Department of Hematology and Oncology, National Children’s Medical Center, Children’s Hospital of Fudan University, Shanghai, China; ^2^ National Children’s Medical Center and the Shanghai Key Laboratory of Medical Epigenetics, Institute of Pediatrics, Institutes of Biomedical Sciences, Children’s Hospital of Fudan University, Fudan University, Shanghai, China

**Keywords:** acute megakaryoblastic leukemia, single cell RNA sequencing, intratumoral heterogeneity, non-DS-AMKL, RACK1, CD81

## Abstract

Pediatric acute megakaryoblastic leukemia (AMKL) is a subtype of acute myeloid leukemia (AML) characterized by abnormal megakaryoblasts, and it is divided into the AMKL patients with Down syndrome (DS-AMKL) and AMKL patients without DS (non-DS-AMKL). Pediatric non-DS-AMKL is a heterogeneous disease with extremely poor outcome. We performed single-cell RNA sequencing (scRNA-seq) of the bone marrow from two *CBFA2T3-GLIS2* fusion-positive and one *RBM15-MKL1* fusion-positive non-DS-AMKL children. Meanwhile, we downloaded the scRNA-seq data of normal megakaryocyte (MK) cells of the fetal liver and bone marrow from healthy donors as normal controls. We conducted cell clustering, cell-type identification, inferCNV analysis, Gene Ontology (GO), Kyoto Encyclopedia of Genes and Genomes (KEGG) enrichment, and Monocle2 analysis to investigate the intratumoral heterogeneity of AMKL. Using canonical markers, we identified and characterized the abnormal blasts and other normal immune cells from three AMKL samples. We found intratumoral heterogeneity of AMKL in various cell-type proportions, malignant cells’ diverse copy number variations (CNVs), maturities, significant genes expressions, and enriched pathways. We also identified potential markers for pediatric AMKL, namely, *RACK1*, *ELOB*, *TRIR*, *NOP53*, *SELENOH*, and *CD81*. Our work offered insight into the heterogeneity of pediatric acute megakaryoblastic leukemia and established the single-cell transcriptomic landscape of AMKL for the first time.

## Introduction

Acute megakaryoblastic leukemia [AMKL or AML-M7 in the French–American–British (FAB) classification] (1) is a subtype of acute myeloid leukemia (AML) characterized by abnormal megakaryoblasts that express platelet-specific surface glycoprotein, including CD41, CD42, and CD61. AMKL is significantly more frequent in children than in adults. In contrast to AMKL in pediatric patients with Down syndrome (DS-AMKL), AMKL in pediatric patients without DS (non-DS-AMKL) is much rarer and more heterogeneous, a significant proportion of whom carry chimeric oncogenes including *RBM15-MKL1*, *CBFA2T3-GLIS2*, *NUP98-KDM5A*, and *MLL* gene rearrangements. *CBFA2T3-GLIS2* is the most frequent chimeric oncogene identified in non-DS-AMKL patients, resulting from a cryptic inversion on chromosome 16 [inv (16) (p13.3q24.3)], which correlates with a poor prognosis (2). *RBM15-MKL1* fusion was identified specifically among infants owing to t(1;22) translocation (3, 4).

Unfortunately, the outcome of non-DS-AMKL is extremely poor, with significantly lower event-free survival than DS-AMKL and pediatric AML, even after undergoing intensified treatment, and its pathological mechanisms had remained elusive till now. Most previous studies were transcriptional profiling of AMKL, which undermined the heterogeneity of tumors. Recently, single-cell RNA sequencing (scRNA-seq) was performed in numerous cancers to study the intratumoral heterogeneity, developing trajectory, and immune microenvironment (5, 6).

Therefore, we conducted scRNA-seq to illuminate molecular features of AMKL for the bone marrow from three AMKL pediatric patients with either *CBFA2T3-GLIS2* fusion or *RBM15-MKL1* fusion and downloaded the single-cell RNA-sequencing data of normal megakaryocyte (MK) cells of the fetal liver and bone marrow from Gene Expression Omnibus (GEO) as normal controls. Then, we analyzed the characteristics of malignant AMKL cells of AMKL that may help us better understand the pediatric non-DS AMKL.

## Materials and methods

### Patient

Three patients diagnosed with AMKL were recruited from the Children’s Hospital of Fudan University in the Department of Hematology and Oncology, including 18-month-old male, 19-month-old female, and 49-month-old female patients. This study was approved by the Medical Ethics Committee of the Children’s Hospital of Fudan University institutional review board and conducted under the Declaration of Helsinki principles (approval reference no. 366). Informed written consent was obtained from the parents before bone marrow biopsy in the study.

### Healthy donors

Healthy donors’ data were downloaded from the GEO dataset (accession number GSE144024). Fetal liver (FL) samples were obtained from healthy donors with elective medical abortion after 4–10 weeks post-conception (WPC). The mononuclear cells of the fetal liver were isolated from human FL and transferred into Iscove’s modified Dulbecco’s medium (IMDM) (GIBCO, CA, United States) containing 10% fetal bovine serum (FBS) (Hyclone, UT, United States), from which the megakaryocytes were sorted by FACS according to CD41a+CD42b+ phenotype.

The scRNA-seq data of the bone marrow was downloaded from the National Omics Data Encyclopedia dataset (NODE; OEP000756, OEP001150, and OEP001128). First, the reporters built a model of megakaryopoiesis *in vitro*. Second, they harvested the cultured cells at the time points of D0, D4, 48, and D12. Lastly, they performed scRNA-seq on 10x genomics platform and filtered out low-quality cells.

### Single-cell RNA sequencing

Experimental procedures followed established techniques using the Chromium Single Cell 3′ Library V3 kit (10x Genomics). In brief, the mononuclear cells obtained from the bone marrow by density gradient centrifugation using lymphocyte separation medium were loaded into the chromium instrument (10x Genomics), and the resulting barcoded cDNAs were used to construct libraries. Raw sequence data were converted into FASTQs using the Illumina bcl2fastq software. FASTQ files were aligned to the human genome (GRCh38) using the CellRanger 4.0 (10x Genomics) pipeline according to the manufacturer’s instructions.

### Quality control

Initial data processing of scRNA-seq for the bone marrow from patients, including A330 (n = 8,092), M002 (n = 8,151), and M704 (n= 8,144) were performed using R4.0.4 with R package Seurat (v4.0.4). Healthy donors’ scRNA-seq data including FL and hiBM were processed following method used by the authors. Individual cells were filtered based on the total number of genes expressed and the percentage of mitochondrial reads. The cells were included with genes >200 but <6,000 and the percentage of mitochondrial reads <10%. Genes that were expressed in fewer than three cells were also removed. For each cell, the expression of each gene was normalized to the sequencing depth of the cell, scaled to a constant depth (10,000), and log-transformed. Variable genes were selected (selection.method= “vst,” nfeatures=2,000, default settings otherwise). Principal component analysis (PCA) was performed on the variable genes, and the optimal number of principal components (PCs) for each sample was chosen using elbow plots (A330, 15; M002, 20; and M704, 15). PCs were used for dimensionality reduction if they were statistically significant according to the elbow plots. Dimensionality reduction and visualization were performed with the Uniform Manifold Approximation and Projection (UMAP) algorithm (Seurat implementation) using the PCs selected above. Unsupervised graph-based clustering of cells was performed using the indicated PCs, and resolution was chosen concerning the cell numbers (A330 = 0.8, M002 = 0.6, and M704 = 0.6).

### AMKL cell identification

The FindAllMarkers function implemented in Seurat was used to identify differentially expressed genes (DEGs) between different clusters. The Wilcoxon test was performed on each gene, and the p-value and adjusted p-value standing for statistical significance were computed. Genes with adjusted p-values <0.05 were considered significant. Cell-type inference was performed in an unsupervised manner by the R package SingleR (v1.4.1). The identification of clusters was accomplished by the canonical marker genes combined. The blast cells were selected, and the inferCNV analysis was performed with parameters including default settings to ascertain these tumor cells by the R package infercnv (v1.6.0).

### Trajectory analysis

The data generated from the tumor cells of specimens A330, M002, and M704 were integrated, and batch effects were corrected by the R package harmony (v1.0). Then, trajectory analysis was performed using the R package Monocle2 (v2.18.0).

### Integration sample analysis

The data generated from the tumor cells of specimens A330, M002, and M704 and normal megakaryocytes from the bone marrow (BM) mentioned before was integrated, and batch effects were corrected by the R package harmony (v1.0). Furthermore, FindMarkers function was used to identify DEGs between tumor cells and normal megakaryocytes. The Wilcoxon test was performed on each gene. The novel markers utilized a default threshold of 4 for the average fold change, 0.01 for the adjusted p-value, and a filter for the minimum delta percent of cells ([X (percentage of cluster1) − X (percentage of cluster2)]/X (percentage of cluster1) × 100) >90%. Meanwhile, the novel markers must be expressed in every AMKL sample.

The DEGs obtained were subjected to GO and KEGG analyses using the R package clusterProfiler (v3.18.1).

### Detection of fusion genes

The reverse transcription polymerase chain reaction (RT-PCR) was used to screen for *CBFA2T3-GLIS2* and *RBM15-MKL1* transcripts. The total RNA was extracted from the bone marrow using Qiagen whole-blood total RNA extraction kit. The purity and concentration of RNA were analyzed. One microgram of total RNA was taken to synthesize cDNA using Thermo’s M-MLV reverse transcriptase and Thermo’s Random 6 MERS (reverse transcriptionist primers) (37°C, 1 h). One microliter (100 ng/μl) of the synthesized cDNA was used to amplify the 75-bp target fusion gene using Thermo Platinum™ II hot-start PCR premix (2X) and *CBFA2T3-GLIS2* or *RBM15-MKL1* primers. The amplified product was taken for agarose gel electrophoresis, and the target fragment with the size of 135 bp was recovered. The amplified products were sequenced using an ABI 3500DX sequencer to analyze the fusion sites. The following primers were used: forward 5′-CGAAGGGCCTCAGCTAGACGT-3′ and reverse 5′-AGCCACTGCGCTATTTGGAT-3′ (*CBFA2T3-GLIS2* fusion); forward 5′-AGCAGTTCCTGGATTCCCCT-3′ and reverse 5′-AAA TGCGGCTGGACTTTT-3′ (*RBM15-MKL1* fusion).

## Results

### A single-cell map of AMKL

These AMKL patients (**Table 1**) comprised one 18-month-old male, one 19-month-old female, and one 49-month-old female patients. The bone marrow samples were collected at the onset of the disease and went through scRNA-seq in the 10x genomics platform. We obtained 24,387 cells after quality control. The batch effect was removed by harmony package.

**Table 1:** The clinical characterizations of the three non-DS-AMKL children.

		Patient	
Feature	A330	M002	M704
Age	18 months	19 months	49
Gender	Male	Female	Female
Fever Hepatomegaly Splenomegaly	+ − −	+ + +	+ + +
WBC (×10^9^/L) HB (g/L) PLT (×10^9^/L) LDH (IU/L)	9.7 84 473 579	6.5 80 37 917	6.8 68 40 5557
%Blasts in bone marrow smear	65	84	23
Karyotype FISH	46, XY[20] nuc ish (EGR1,D5S23, D7S486,D7Z1,CBFB,ETO, AML1,MLL,PML, RARA, BCR, ABL)[200]	46, XX[20] nuc ish (EGR1,D5S23, D7S486,D7Z1,CBFB,ETO, AML1,MLL,PML, RARA, BCR, ABL)[200]	59, XX, t(1;22)(p13.3;q13),+der(1)t(1;22)x2,+2,+4,+5,+6,+10,+18,+19,+19,+20,+21,+22[15]/59,sdl,+del(8)(p11.2),-21[3]/59,sdl,t(3;3) (p26;q21)[2] ISCN:nuc ish (EGR1,D5S23)X3[100/200],(ETOx2,AML1x3)[90/200]/(ETOx3,AML1x2)[20/200],(D7S486,D7Z1,MLL,PML,RARA,CBFB)[199]
Fusion genes	*CBFA2T3-GLIS2* (+)	*CBFA2T3-GLIS2* (+)	*RBM15-MKL1* (+)
Gene mutation	NBAS: p. Q196X	NA	*RUNX1*: p.M466L; *CEBPA; TET2; ASXL1*
CD33 CD34 CD41 cyCD41 CD56 HLA-DR CD38	+ + + - + − −	+ + + + + − −	+ − + + − − +
Prognosis	CR	CR	CR

PB, peripheral blood; WBC, white blood cell; N, neutrophil; HB, hemoglobin; PLT, platelet; CRP, C-reaction protein; LDH, lactate dehydrogenase; CR, complete remission.

**Figure 1:**
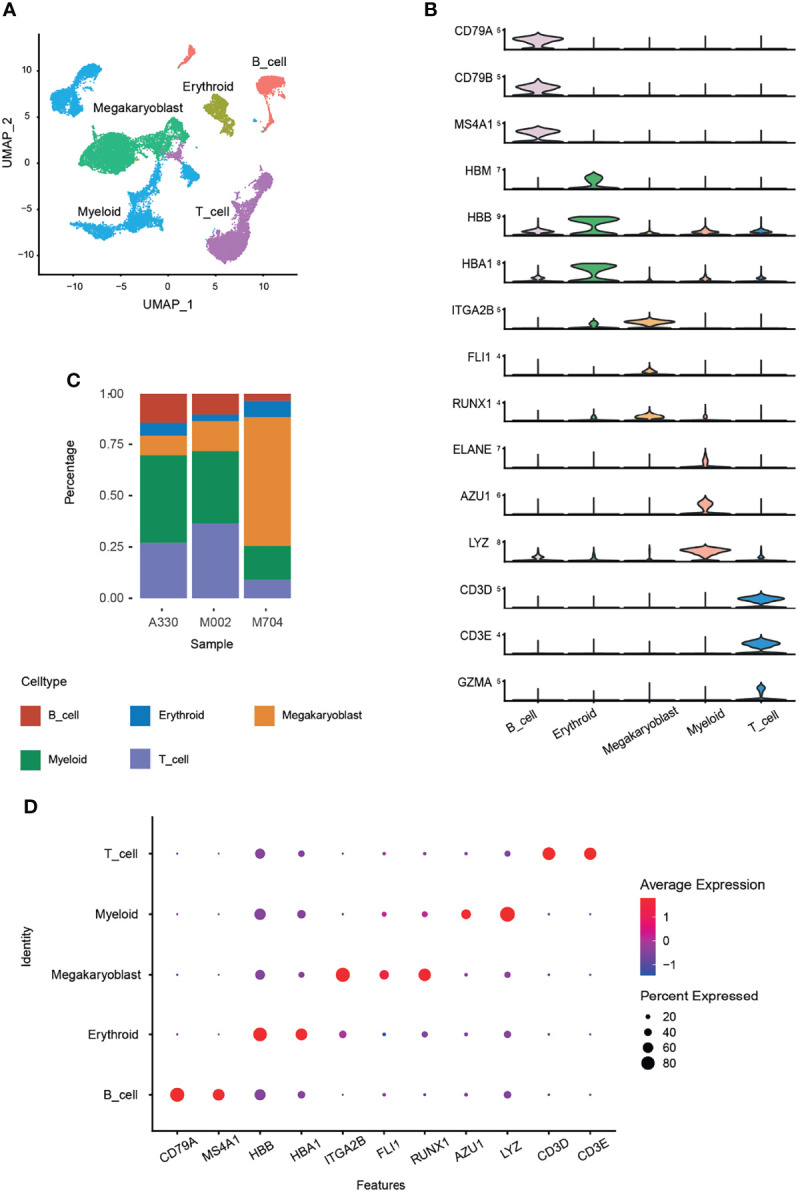
Identification of cell types using Seurat and Harmony packages. The bone marrow cells from three AMKL children were performed by scRNA-seq of the 10x genomics platform. A total of 24,387 cells were obtained after quality control. The batch effects were removed using the Harmony package. **(A)** The uniform manifold approximation and projection (UMAP) plot of the main cell types in AMKL samples. **(B)** The normal cell types were identified by classical marker genes, and the malignant cells, i.e., megakaryoblasts, were considered by canonical markers of megakaryoblastic lineage. **(C)** The proportion of the main cell types was calculated in each sample. **(D)** The dot plots show the average expression levels of signature genes in the main cell types.

Five main cell types were identified by canonical marker genes (**Figure 1A**). For example, *CD79A*, *CD79B*, and *MS4A1* for B cells; *HBB*, *HBA1*, and *HBM* for erythrocytes; *CD3D*, *CD3E*, and *GZMA* for T cells; *ELANE*, *AZU1*, and *LYZ* for myelocytes. Signature markers, namely, *ITGA2B* (CD41), *FLI1*, and *RUNX1*, were specifically and highly expressed in abnormal megakaryoblasts (**Figure 1B**).

To explore the intratumoral heterogeneity of AMKL, we compared the proportion of the cell types (**Figure 1C**) and found out that M704 had the highest proportion and number of megakaryoblasts, while A330 had the least. Interestingly, the T and B cells had an inverse outcome, with a significant higher composition in M002 and A330 than in M704. The intratumoral heterogeneity in the percentage of myeloid cells is in accordance with T and B cells. The myeloid cells incorporate granulocyte–monocyte progenitor cells (GMPs) and monocytes. The dot plot exhibited the typical high average expression and percent expressed in every cell identity explicitly (**Figure 1D**).

### The intratumoral heterogeneity of malignant AMKL cells

**Figure 2:**
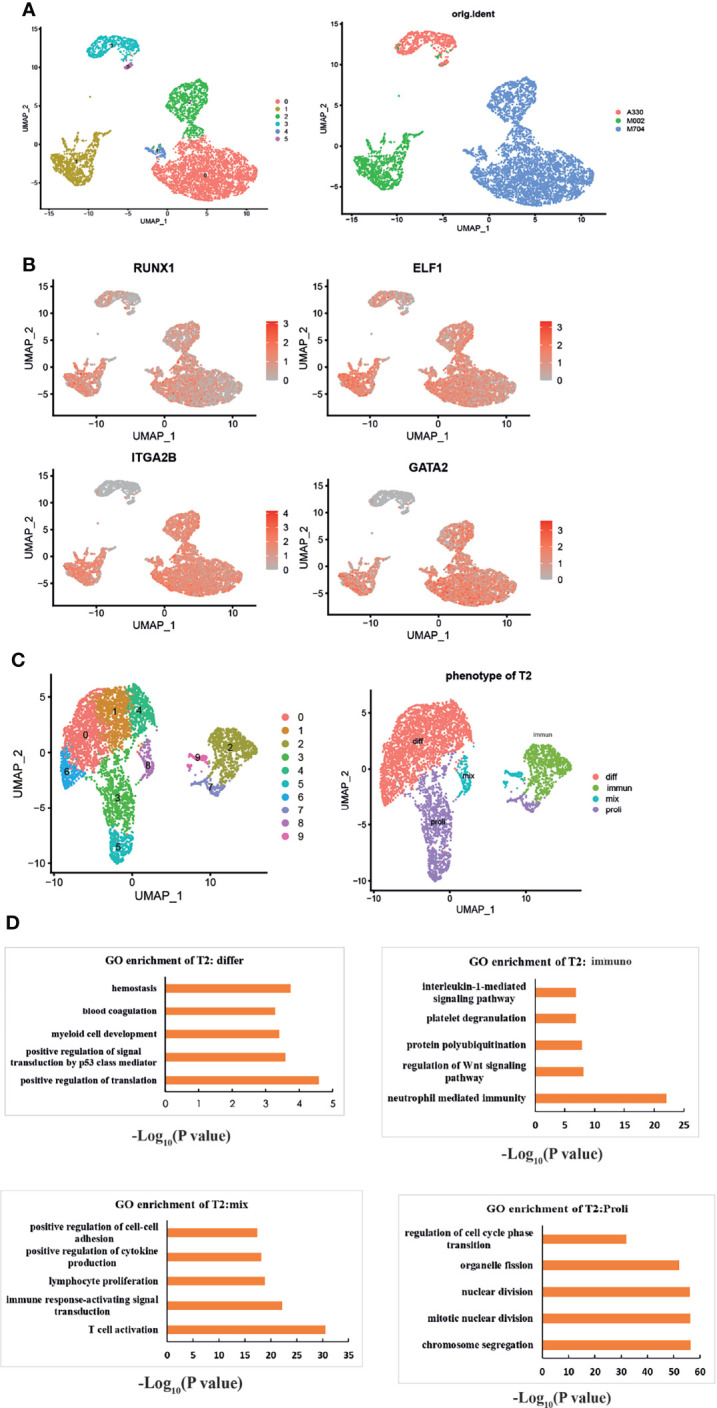
The intratumoral heterogeneity of abnormal megakaryoblasts. **(A)** The UMAP plots show six subclusters in the three samples. **(B)** UMAP plots showing the expression of the signature genes following the MK lineage. The distinct malignant cell types of immature-like T1 cells (highly expressed MK’s markers but not mature markers like ITGA2B) and mature-like T2 cells (highly expressed all MK’s markers especially mature markers like ITGA2B) were identified. The red color means the genes are highly expressed, and gray means low or not expressed. **(C)** UMAP visualization of four different phenotypes among T2. “Differ” refers to differentiation, “immune” refers to immunity, “mix” refers to mix lineages, and “proli” refers to proliferation. **(D)** The GO enrichment plots of each phenotype of T2. All enriched pathways and biological processes are under a p-value of 0.05 and correction of FDR.

We obtained the subset of malignant AMKL cells. Generally, six clusters were identified based on the UMAP analysis (**Figure 2A**). Clusters 0, 2, and 4 were included in sample M704. Cursorily, cluster 1 was close to M002. Clusters 3 and 5 constituted sample A330. The clusters that belonged to different samples were far away from each other.

Through the expressions of specific megakaryocytic lineage marker genes and some highly expressed genes in MKs, we explored the intratumoral heterogeneity of AMKL stepwise (**Figure 2B**). *RUNX1* and GATA2 are essential for normal megakaryopoiesis (7). *ELF1* (E74-like ETS transcription factor 1) is one the top 10 transcriptional factors (TFs) among MKs (5) in yolk sac (YS), which warrants further research. Experiments showed that platelet factor 4 (*PF4*), an inhibitor of megakaryopoiesis (8), is regulated by *ELF1* (9). Interestingly, nearly all tumor clusters were highly expressing *RUNX1* and *ELF1*, but the clusters that belonged to M002 and M704 had high expression of *ITGA2B* and *GATA2*. Therefore, we termed the tumor clusters 3 and 5 as T1 and clusters 0, 1, 2, and 4 as T2.

To have a deeper understanding of the heterogeneity in T2, we identified 10 subclusters through UMAP analysis (**Figure 2C**). Owing to signature genes in the top 10 DEGs of the clusters, we identified four major types of T2 (**Figure 2C**). For example, the expression of cell-cycle-associated genes of *MCM5*, *TYMS*, *TOP2A*, *MKI67*, *UBE2C*, and *TUBB4B* in clusters 3, 5, and 7 indicated the proliferating T2 cells. The clusters 3, 5, and 7 were termed as “Proli” then. In particular, the clusters 8 and 9 expressed many myeloid or lymphoid markers, such as *LYZ*, *S100A9*, *IGLC2*, and *CD74*. Clusters 8 and 9 were termed as “mix” population hereafter. In addition, clusters 0 and 6 expressed HBD, indicating for MK-erythroid progenitor (MEP)-like AMKL cells, and clusters 1 and 4 highly expressed the ribosomal genes, suggesting active protein synthesis progress. Then, we termed clusters 0, 1, 4, and 6 as “diff” cells (differentiated cells). The remaining cluster 2 was identified as “immun” (immunological cells) by the GO enrichment analysis (**Figure 2D**). Intriguingly, the enriched pathways in the immunological T2 cells were interleukin-1 (IL-1)-mediated signaling pathway, neutrophil-mediated immunity, regulation of Wnt signaling pathway, and protein polyubiquitination. In particular, the enrichment of the four subpopulations further consolidated our findings (**Figure 2D**). The “diff” subcluster was enriched in hemostasis, blood coagulation, and myeloid development. The proliferating subcluster exhibited regulation of cell cycle phase transition and nuclear division, while the “mix” malignant cells enriched in T-cell activation, positive regulation of cytokine production, and upregulation of cell–cell adhesion by the GO enrichment analysis.

We tried to identify the *CBFA2T3-GLIS2* fusion-positive subpopulation by the immunotyping of bright CD56 (encoded by *NCAM1*) and negative HLA-DR according to a report (10) (**Supplementary Figures S1A**, **B**). Unfortunately, our results did not seem fully coordinated with the study, as M002 (mainly cluster 1) and A330 (including cluster 3,5) are both *CBFA2T3-GLIS2* fusion positive, but had an opposite immunophenotype in terms of CD56 and HLA-DR.

### The copy number variations of malignant AMKL cells

**Figure 3:**
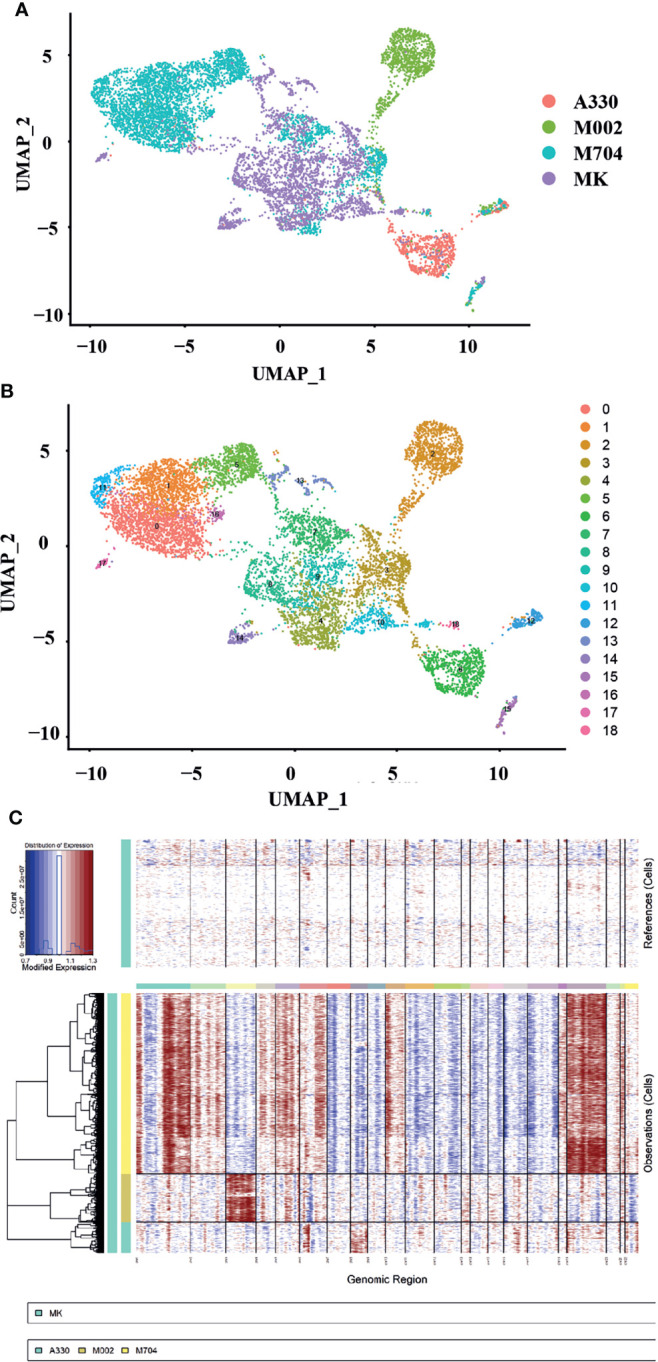
The single-cell landscape and copy number variance (CNV) speculation of the tumors and normal MK cells of the fetal liver (FL). **(A, B)** UMAP plots showing the single-cell landscape of tumor cells in AMKL and normal MK cells in FL (the control). **(C)** The plot shows the CNV levels of AMKL tumor and the normal control. The upper column represents the CNVs of normal FL MK cells. The x-axis exhibits chr1 to chr22. The y-axis exhibits the cell clusters. The red color represents the gain of copies, and blue represents loss of copies.

**Figure 4:**
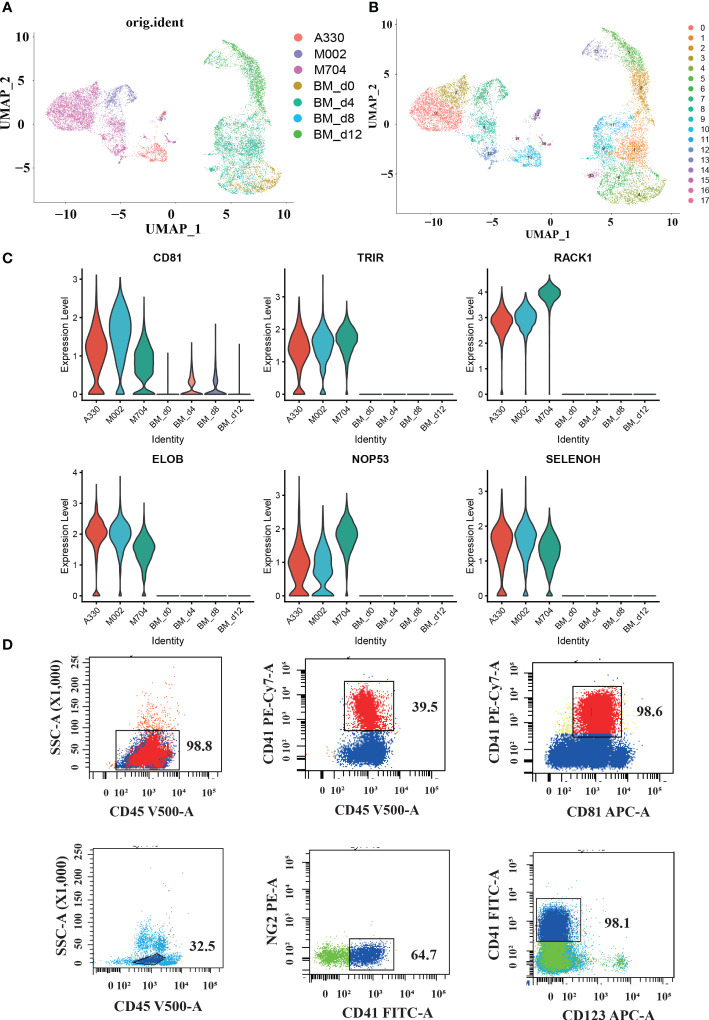
The single-cell profiling of tumor cells in AMKL and normal MK cells in the bone marrow (BM). **(A, B)** UMAP plots of the combination of malignant AMKL cells and normal MK cells in BMs. **(C)** Violin plots showing candidate marker genes in abnormal megakaryoblasts. **(D)** Surface immunophenotype of another AMKL patient.

We downloaded the single-cell transcriptional data for normal megakaryocytes (MKs) control from GEO (GSE144024) dataset. We analyzed the scRNA-seq data for FL following Zhou’s instructions (11). The results were in concert with Zhou’s figures. Then, we pooled the MKs, MK-erythroid-mast cell progenitors (MEMPs), and hematopoietic stem and progenitor cells (HSPCs) together with UMAP analysis. For further analysis, we collected MKs and MEMPs as the megakaryocytic cells and named them after MK instead. Then, we merged the malignant cells from A330, M002, and M704, with MK mentioned before (**Figure 3A**). Using UMAP analysis, 19 unbiased clusters were obtained (**Figure 3B**). Interestingly, the normal MK population from FL had overlaps with the abnormal megakaryoblastic cells, especially the ones from the M704 sample. Nevertheless, the normal megakaryocytic cells from the BM had little connection with malignant AMKL cells through UMAP analysis (**Figure 4A**). It can be postulated that the malignant AMKL cells had more similarities with immature MK cells from FL than BM.

To explore the copy number variations (CNVs) of abnormal AMKL cells, the inferCNV package was applied when using MK from FL as normal control (**Figure 3C**). The clonality analysis of abnormal megakaryoblastic cells placed the intratumoral heterogeneity into spotlight. Corresponding to the patients’ fluorescence *in situ* hybridization (FISH) analysis of chromosomes, the M704 had the most complexed CNVs (**Supplementary Material**, Png.S1, PPT.S1). The sample M704 gained a copy number of whole chromosomes 19 and 21 and large regions of chromosomes 1, 2, 4, 5, 6, 10, 18, and 20 while loss regions of chromosomes 7, 8, 9, 11, 12, 13, 14, 15, 16, and 17. For M002, an integrated copy number of chromosome 3 was acquired, and a nearly complete copy number of chromosome 8 was gained in A330. Some regions of chromosomes 1, 4, 5, 6, 12, 16, and 19 were gained in M002, and little parts of chromosomes 1, 2, 6, 14, 16, 17, 19, 20, and 22 were received within A330. Despite that the findings of aberrant chromosomes in A330 and M002 by FISH analysis seemed to be clear, which contradicts the clonality analysis, it might elucidate a higher accuracy of CNVs. In summary, M704 had much more complexing CNVs than A330 and M002 based on the FISH findings.

### The potential markers of AMKL

In order to explore the latent signature markers of AMKL, we combined our data with the downloaded scRNA-seq data of MK from BM (**Figures 4A**, **B**). First, we downloaded the data of hiBM (*in vitro* megakaryopoiesis model of BM) from National Omics Data Encyclopedia (NODE): OEP000756, OEP001150, and OEP001128. The time points of hiBM are D0, D4, D8, and D12. Then, we identified megakaryocytic cells through UMAP visualization and marker recognition. The megakaryocytic cells include MKs, MK progenitors (MKPs), MEPs, and immature MEPs (namely, MEP2s). As mentioned before, the abnormal AMKL cells are apart from the normal MKs. In particular, the A330 with least CNVs seems to be closer to the normal MKs.

Furthermore, we conducted comparisons between malignant AMKL cells and normal megakaryocytic cells to find some potential markers. The violin plots showed extraordinarily high expression of *RACK1*, *ELOB*, *TRIR*, *NOP53*, *SELENOH*, and *CD81* in abnormal megakaryoblastic cells exclusively (**Figure 4C**). In addition, the normal megakaryocytic cells are confirmed with canonical genes by violin plots.

Studies have shown the diverse expression of *CD81* in *de novo* AML and considered high expression of *CD81* as a sign of poor prognosis (12). Meyling’s work enrolled FAB types M0, M1, M2, M4, M5, and M6, but not M7. Therefore, our study filled this up with the exclusively high expression of *CD81* in the malignant cells of three AML-M7 (AMKL) BM samples through scRNA-seq analysis. Maybe, *CD81* has relationships with the adverse prognosis in non-DS-AMKL. We also performed flow cytometry on another pediatric non-DS-AMKL; *CD81* expression in the AMKL blasts were nearly 100% (98.6% for sample ZYL, **Figure 4D**).

**Figure 5:**
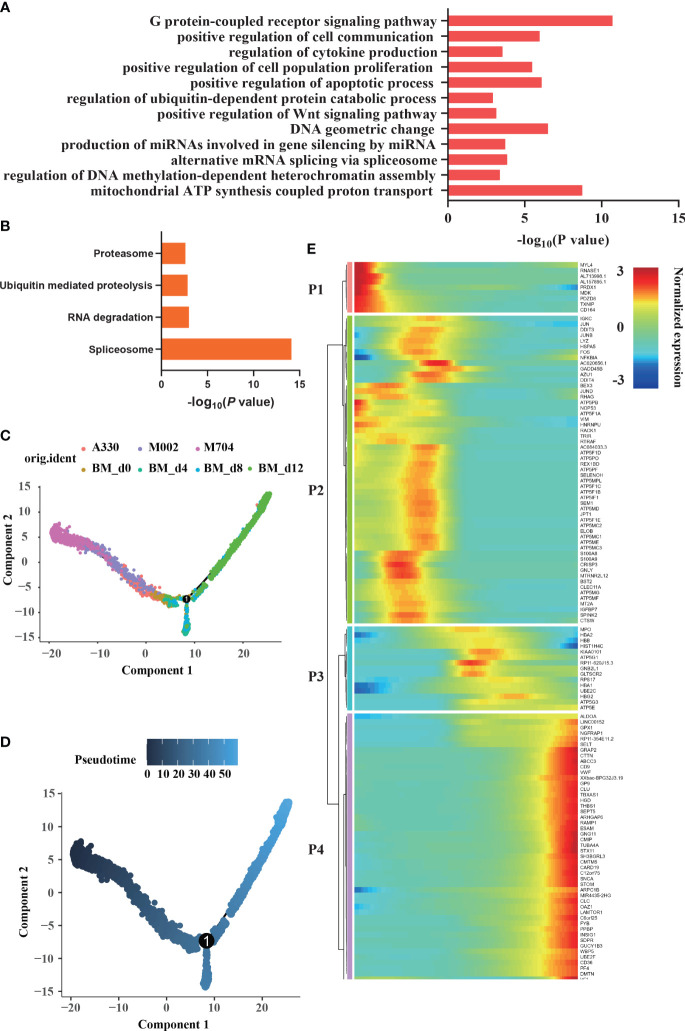
Enrichment and trajectory analyses of abnormal megakaryoblasts in AMKL and normal MK cells. **(A, B)** The enrichment of GO and KEGG of differentially expressed genes (DEGs) between megakaryoblasts in AMKL and normal MKs separately. **(C, D)** The trajectory analysis of megakaryoblasts in AMKL and normal MKs. The MKs (hiBM) were chosen from a human *in vitro* megakaryopoiesis model with time points of d0, d4, d8, and d12. **(E)** The heatmap showing the normalized expression of four gene sets naming P1, P2, P3, and P4. P1 stands for the DEGs highly expressed in tumors, while P4 stands for the ones highly expressed in normal mature MKs.

The enrichment analysis of malignant cells showed G-protein-coupled receptor signaling pathway, regulation of DNA methylation-dependent heterochromatin assembly, alternative mRNA splicing *via* spliceosome, regulation of ubiquitin-dependent protein catabolic process, regulation of cytokine production, and positive regulation of cell communication (**Figure 5A**). According to KEGG analysis (**Figure 5B** ), RNA degradation, ubiquitin-mediated proteolysis, proteasome, and spliceosome were enriched in abnormal AMKL cells. Undoubtedly, the normal megakaryocytic clusters are enriched in positive regulation of blood coagulation, platelet aggregation, and platelet activation.

### Trajectory analysis of malignant AMKL cells

To further understand the difference of AMKL, trajectory analysis was performed (**Figures 5C**, **D**). Particularly, the abnormal AMKL cells were the root locus, which were even more primitive than the immature MEPs (**Supplementary Figure S2B**). Despite the MEPs bifurcated into megakaryocytic differentiation and erythroid-primed differentiation as time went by, M704 were the most primal cells at the beginning; meanwhile, A330 were the most differentiated cells shifted into the normal ones (**Supplementary Figure S2A**). Interestingly, M002 seemed to be the transition state of M704 and A330. As shown in the heatmap plot (**Figure 5E**), the gene set P1 enriched in malignant cells incorporate *MDK*, which is involved in the pathway of Nanog in mammalian ESC (embryonic stem cell) pluripotency. Indeed, *NANOG* plays a vital role in CSCs (cancer stem cells). *MYL4* and *PDZD8*, the cytoskeleton-associated genes in P1, were inferred to the hyperproliferation of malignant cells. The immunology-related genes, namely, *TXNIP* and *CD164*, were also shown in P1. The findings of malignant cells highly confirmed our former discoveries.

The gene set P3 including genes *HBB*, *HBA2*, and *HBA1* reinforced that the bifurcation was erythroid-primed differentiation. The genes like *CD9*, *VWF*, *THBS1*, *PF4*, and *PPBP* were enriched in the gene set P4, indicating MKs. The gene set P2 seemed to be in the middle of the root locus and normal MEPs. Strikingly, plenty of ATP-related genes and some proto-oncogenes (*JUN*, *JUND*, and *FOS*) were enriched in P2. The potential markers except *CD81* were found in P2, which hinted that the potential markers had something to do with the immaturity of malignant cells. *CD81* may be excluded from P2 because of the strict criterion set.

**Figure 6:**
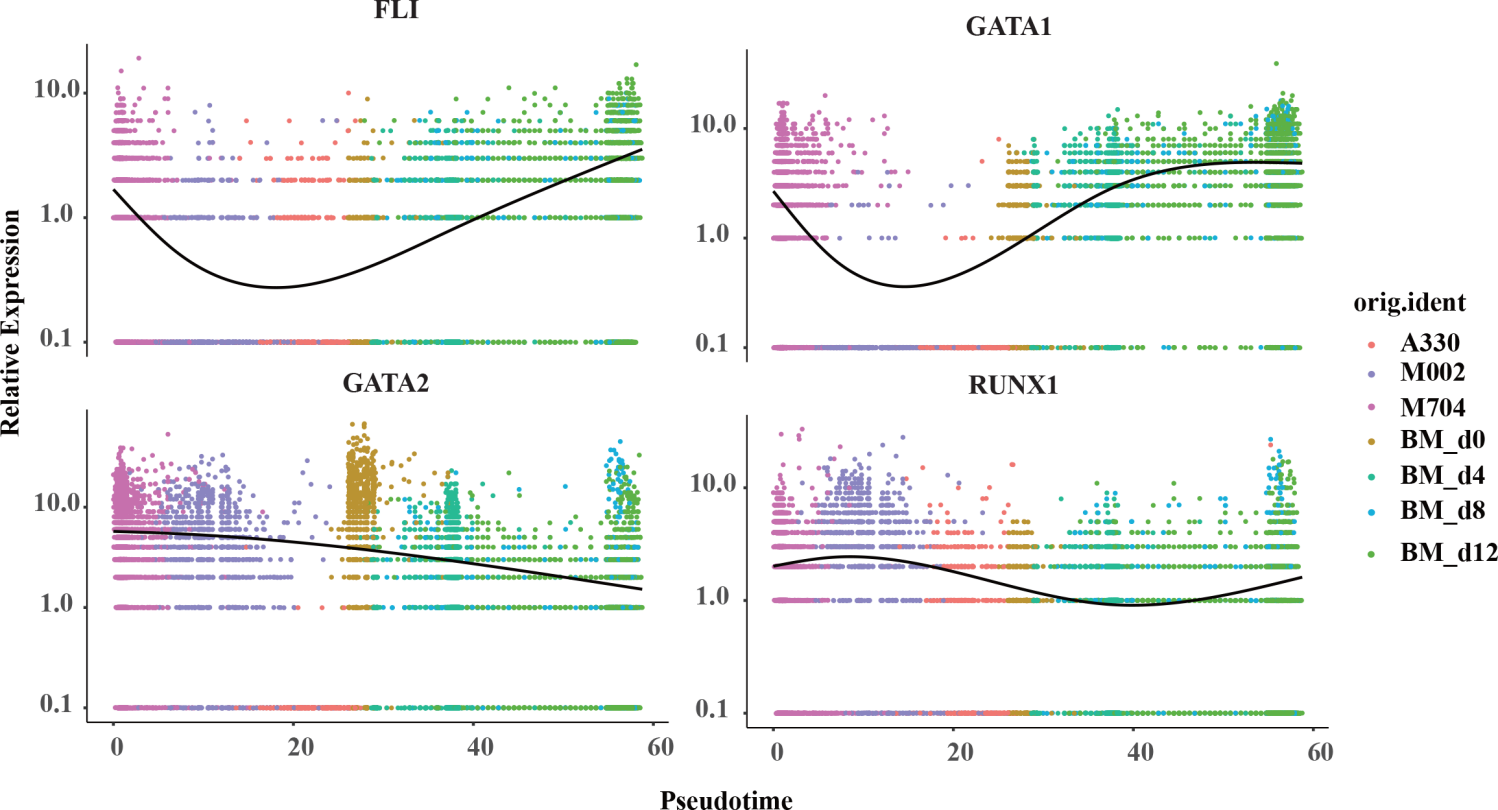
The pseudotime curve of the crucial signature genes in megakaryopoiesis. The pseudotime curve shows the transformation of marker genes FLI1, GATA1, GATA2, and RUNX1 in the combination of tumors in AMKL and normal MKs in hiBMs.

Aiming to have a deeper understanding of the crucial genes in the pseudotime trajectory, the curves were created in the plots (**Figure 6**; **Supplementary Figures S3A**, **B**). Surprisingly, the expression of *FLI1* and *GATA1* struck high in the start point of M704 and reached bottom in A330. However, the curve for *GATA2* was descending smoothly. The expression of *RUNX1* was likely to have the least variations. Overall, T1 (namely the blasts of A330) seemed to be more mature than T2 (malignant cells of M704 and M002).

## Discussion

The acute megakaryoblastic leukemia without DS syndrome is highly heterogeneous with adverse prognosis (13). Although previous studies had found some chimeric oncogenes, such as *CBFA2T3-GLIS2*, *RBM15-MKL1*, and *NUP98-KDM5A* that played an important role in the tumorigenesis and oncogenesis (14–16), the pathological mechanism of non-DS-AMKL remains largely unknown. Herein, our study unveiled the heterogeneity of pediatric non-DS-AMKL and discovered that some markers might be drug targets by single-cell analysis. The intratumoral heterogeneity of AMKL was characterized with the diverse cell-type proportions, expressions of signature genes, enriched pathways, CNVs, trajectory, and immaturity of abnormal cells in different samples. The markers detected in the malignant AMKL cells including *RACK1*, *ELOB*, *TRIR*, *NOP53*, *SELENOH*, and *CD81* might have relationships with the tumorigenesis or be therapeutic targets. Therefore, our study may provide insights for a deeper understanding of non-DS pediatric AMKL.

We dissected the single-cell atlas of AMKL and unveiled the cellular heterogeneity of different samples. M704 had the maximum scale of blasts and minimum scale of lymphocytes. As is shown in **Supplementary Table S1**, A330 and M002 were *CBFA2T3-GLIS2* (i.e., *ETO2-GLIS2*) fusion positive, while M704 was *RBM15-MKL1* (i.e., *OTT-MAL*) fusion positive. As reported, *CBFA2T3-GLIS2* fusion (18.6%) is the most frequent fusion event in pediatric non-DS AMKL cohort alongside with inferior outcome (17). A Children’s Oncology Group (COG) pediatric AML study (16) showed that *CBFA2T3-GLIS2* fusion was cooperated with little recurrent mutations and upregulation of CD56, which was coordinated with our findings. Both A330 and M002 were of normal karyotype. Although A330 had mutations of *NBAS*, it had been reported in recurrent acute liver failure (18) without connection with leukemia so far. A330 and M002 were both 100% positive in CD56, which seemed be to a feasible target of *CBFA2T3-GLIS2* fusion-positive patients as predicted in the report (16). Intriguingly, the reported *RBM15-MKL1* fusion was found only in infant AMKL (3), which is in disagreement with the non-AMKL case M704, whose age was 49 months old at the onset of disease.

To further understand the difference of the disease, we separated the malignant cells from the normal ones and discovered two subtypes termed as T1 and T2. T2 mainly includes M704 and M002, while T1 contains A330. T2 had a higher expression of *GATA2*, *FLI1*, *GATA1*, and *ITGA2B* than T1 but similar expression of *RUNX1* and *ELF1*. Later, we found that T1 is more mature than T2 by Monocle2 algorithm. The findings were meaningful because the expression of *GATA1* and *ITGA2B* was considered to rise while *GATA2* and *FLI1* to descend as the megakaryocyte differentiated (19). One guess of this paradox was the difference in fusion genes between T1 and T2. Maybe, *CBFA2T3-GLIS2* fusions have stronger power to unbalance the relative expressions of *GATA2* and *GATA1* than *RBM15-MKL1* fusions (4, 15). Thirant et al. (15) showed that *CBFA2T3-GLIS2* oncogene can cause AMKL by upregulating *ERG* and downregulating *GATA1* strikingly. On the other hand, *RBM15-MKL1* activates RBPJ and MPL to induce AMKL as reported by Mercher et al. (20). However, it can hardly explain the expressions of *RUNX1* and *FLI1*. Thus, a higher possibility might be the intratumoral heterogeneity due to abnormal megakaryopoiesis. As we know, *GATA2* was downregulated while *GATA1* was upregulated in normal megakaryopoiesis. However, it was discordant in the malignant AMKL cells and normal counterparts as shown in **Figure 6**. The changing expressions of *RUNX1* and *FLI1* showed a similar phenomenon, in which malignant cells do not mature as with the normal ones. This hinted that considering only one canonical marker such as *GATA1* to be the measure of maturity in abnormal cells can lead to wrong answers.

The infercnv algorithm seemed to perform well in analyzing the CNVs of AMKL. The results kept up with the observation in FISH. It can be envisioned that M704 were in an aggressive state at the top of megakaryocytic differentiation with the most CNVs, M002 were the intermediate state, and A330 were the differentiated ones. Nevertheless, utilizing immunotyping of *CBFA2T3-GLIS2* as in previous report (10) did not work out well. M002 with *CBFA2T3-GLIS2* fusions is identical to the definition with positive CD56 and negative HLA-DR and CD45, but is reversed A330. Because our sample numbers are limited, the results may need further consolidation. One guess is that the heterogeneity of AMKL is far more complexed than that of other AMLs; thus, the report may not have taken into account enough non-DS AMKL.

Furthermore, we tried to find markers between malignant AMKL cells and normal MKs in the bone marrow. The markers, including *RACK1*, *ELOB*, *TRIR*, *NOP53*, *SELENOH*, and *CD81*, are exclusively expressed in abnormal counterparts, which might provide clues for the tumorigenesis and therapeutic targets. *CD81* was found within blasts in comparison with that in fetal liver and other cell types. The flow cytometry results showing high expression of *CD81* in AMKL blasts suggest a potential marker for AML-M7. Telomerase RNA component interacting RNase (TRIR) encoded by *TRIR* is also known as C19orf43 or fSAP18. TRIR is an exoribonuclease that may have functions in RNA cleavage, cancer suppression, and antiviral activities like other ribonucleases (21, 22), although it remains to be a novel target in tumors with largely unknown functions. The GO enrichment of upregulated genes in AMKL was associated with positive regulation of cell communication, cell proliferation, apoptosis, and Wnt signaling pathway, which were coordinated with Smith’s report (16).

In conclusion, our study provided the single-cell transcriptomic landscape of AMKL, which have not been established till now. We discovered the intratumoral heterogeneity of AMKL in single-cell resolution, which was shown in various cell-type proportions, disparate CNVs, gradual maturity of malignant cells, and varied momentous genes or pathways enrichment in different patients. We also find significant genes like *RACK1*, *ELOB*, *TRIR*, *NOP53*, *SELENOH*, and *CD81*, which might be potential markers and drug targets of AMKL. However, it was only explored in three samples, which were limited by the scarcity of AMKL cases. The potential targets shall be verified after more experiments, especially on *CD81*, which we highly recommend. After all, the non-DS-AMKL still needs further study to improve the clinical outcomes.

## Data availability statement

The original contributions presented in the study are publicly available. The datasets have been deposited in the National Genomics Data Center, accession code: PRJCA010386.

## Ethics statement

The studies involving human participants were reviewed and approved by the Medical Ethics Committee of the Children’s Hospital of Fudan University institutional review board. Written informed consent to participate in this study was provided by the participants’ legal guardian/next of kin. Written informed consent was obtained from the individual(s), and minor(s)’ legal guardian/next of kin, for the publication of any potentially identifiable images or data included in this article.

## Author contributions

XWZ and MQ designed the research and are responsible for this study. NS and JY collected samples and clinical information and analyzed the single-cell RNA sequencing data. XWZ, MQ and NS drafted the manuscript. ZL, HW, YF, XQ, XHZ, HM, YY, WJ and JL provided valuable advice and contributed to sample collection and manuscript revision. All authors contributed to the article and approved the submitted version.

## Funding

The work was supported by the National Natural Science Foundation of China (82141125), the Cyrus Tang Foundation (ZSBK0070), Shanghai Fudan University Education Development Foundation (ZSBK0046), Shanghai Municipal Committee of Science and Technology (19DZ1910602), and Shanghai Hospital Development Center (SHDC12019121).

## Acknowledgments

Thanks for RD and JW's help in sample collection.

## Conflict of interest

The authors declare that the research was conducted in the absence of any commercial or financial relationships that could be construed as a potential conflict of interest.

## Publisher’s note

All claims expressed in this article are solely those of the authors and do not necessarily represent those of their affiliated organizations, or those of the publisher, the editors and the reviewers. Any product that may be evaluated in this article, or claim that may be made by its manufacturer, is not guaranteed or endorsed by the publisher.

## References

[1] Gassmann W Loffler H. Acute megakaryoblastic leukemia. Leuk Lymphoma (1995) 18(Suppl 1):69–73. doi: 10.3109/10428199509075307 7496359

[2] Qi H Mao Y Cao Q Sun X Kuai W Song J. Clinical characteristics and prognosis of 27 patients with childhood acute megakaryoblastic leukemia. Med Sci Monit (2020) 26:e922662. doi: 10.12659/MSM.922662 32532951 PMC7309653

[3] Gruber TA Downing JR. The biology of pediatric acute megakaryoblastic leukemia. Blood (2015) 126(8):943–9. doi: 10.1182/blood-2015-05-567859 PMC4551356 26186939

[4] Masetti R Guidi V Ronchini L Bertuccio NS Locatelli F Pession A. The changing scenario of non-down syndrome acute megakaryoblastic leukemia in children. Crit Rev Oncol Hematol (2019) 138:132–8. doi: 10.1016/j.critrevonc.2019.04.011 31092368

[5] Li Z Wang H Dong R Man J Sun L Qian X. Single-cell RNA-seq reveals characteristics of malignant cells and immune microenvironment in subcutaneous panniculitis-like T-cell lymphoma. Front Oncol (2021) 11:611580. doi: 10.3389/fonc.2021.611580 33816243 PMC8013729

[6] Dong R Yang R Zhan Y Lai HD Ye CJ Yao XY. Single-cell characterization of malignant phenotypes and developmental trajectories of adrenal neuroblastoma. Cancer Cell (2020) 38(5):716–33 e6. doi: 10.1016/j.ccell.2020.08.014 32946775

[7] Li YY Jin C Bai H Gao YX Sun S Chen L. Human NOTCH4 is a key target of RUNX1 in megakaryocytic differentiation. Blood (2018) 131(2):191–201. doi: 10.1182/blood-2017-04-780379 29101237 PMC5757696

[8] Lambert MP Rauova L Bailey M Sola-Visner MC Kowalska MA Poncz M. Platelet factor 4 is a negative autocrine *In vivo* regulator of megakaryopoiesis: Clinical and therapeutic implications. Blood (2007) 110(4):1153–60. doi: 10.1182/blood-2007-01-067116 PMC1976471 17495129

[9] Okada Y Nobori H Shimizu M Watanabe M Yonekura M Nakai T. Multiple ETS family proteins regulate PF4 gene expression by binding to the same ETS binding site. PloS One (2011) 6(9):e24837. doi: 10.1371/journal.pone.0024837 21931859 PMC3171469

[10] Zangrando A Cavagnero F Scarparo P Varotto E Francescato S Tregnago C. CD56, HLA-DR, and CD45 recognize a subtype of childhood AML harboring CBFA2T3-GLIS2 fusion transcript. Cytometry A. (2021) 99(8):844–50. doi: 10.1002/cyto.a.24339 PMC8451792 33811445

[11] Wang H He J Xu C Chen X Yang H Shi S. Decoding human megakaryocyte development. Cell Stem Cell (2021) 28(3):535–49 e8. doi: 10.1016/j.stem.2020.11.006 33340451

[12] Boyer T Guihard S Roumier C Peyrouze P Gonzales F Berthon C. Tetraspanin CD81 is an adverse prognostic marker in acute myeloid leukemia. Oncotarget (2016) 7(38):62377–85. doi: 10.18632/oncotarget.11481 PMC5308734 27566555

[13] Wang Y Lu A Jia Y Zuo Y Zhang L. Outcome and prognostic features in pediatric acute megakaryoblastic leukemia without down syndrome: A retrospective study in China. Clin Lymphoma Myeloma Leuk (2021) 21(4):e301–e8. doi: 10.1016/j.clml.2020.11.001 33257285

[14] de Rooij JD Masetti R van den Heuvel-Eibrink MM Cayuela JM Trka J Reinhardt D. Recurrent abnormalities can be used for risk group stratification in pediatric AMKL: A retrospective intergroup study. Blood (2016) 127(26):3424–30. doi: 10.1182/blood-2016-01-695551 PMC5161011 27114462

[15] Thirant C Ignacimouttou C Lopez CK Diop M Le Mouel L Thiollier C. ETO2-GLIS2 hijacks transcriptional complexes to drive cellular identity and self-renewal in pediatric acute megakaryoblastic leukemia. Cancer Cell (2017) 31(3):452–65. doi: 10.1016/j.ccell.2017.02.006 28292442

[16] Smith JL Ries RE Hylkema T Alonzo TA Gerbing RB Santaguida MT. Comprehensive transcriptome profiling of cryptic CBFA2T3-GLIS2 fusion-positive AML defines novel therapeutic options: A COG and TARGET pediatric AML study. Clin Cancer Res (2020) 26(3):726–37. doi: 10.1158/1078-0432.CCR-19-1800 PMC7002196 31719049

[17] de Rooij JD Branstetter C Ma J Li Y Walsh MP Cheng J. Pediatric non-down syndrome acute megakaryoblastic leukemia is characterized by distinct genomic subsets with varying outcomes. Nat Genet (2017) 49(3):451–6. doi: 10.1038/ng.3772 PMC5687824 28112737

[18] Ricci S Lodi L Serranti D Moroni M Belli G Mancano G. Immunological features of neuroblastoma amplified sequence deficiency: Report of the first case identified through newborn screening for primary immunodeficiency and review of the literature. Front Immunol (2019) 10:1955. doi: 10.3389/fimmu.2019.01955 31507590 PMC6718460

[19] Noetzli LJ French SL Machlus KR. New insights into the differentiation of megakaryocytes from hematopoietic progenitors. Arterioscler Thromb Vasc Biol (2019) 39(7):1288–300. doi: 10.1161/ATVBAHA.119.312129 PMC6594866 31043076

[20] Mercher T Raffel GD Moore SA Cornejo MG Baudry-Bluteau D Cagnard N. The OTT-MAL fusion oncogene activates RBPJ-mediated transcription and induces acute megakaryoblastic leukemia in a knockin mouse model. J Clin Invest (2009) 119(4):852–64. doi: 10.1172/JCI35901 PMC2662544 19287095

[21] Xie J Chen Z Zhang X Chen H Guan W. Identification of an RNase that preferentially cleaves a/G nucleotides. Sci Rep (2017) 7:45207. doi: 10.1038/srep45207 28322335 PMC5359670

[22] Smith EM Pendlebury DF Nandakumar J. Structural biology of telomeres and telomerase. Cell Mol Life Sci (2020) 77(1):61–79. doi: 10.1007/s00018-019-03369-x 31728577 PMC6986361

