# Supplementary material for: Revealing the intratumoral heterogeneity of non-DS acute megakaryoblastic leukemia in single-cell resolution
Source: Front Oncol. 2022 Aug 8;12:915833. doi: 10.3389/fonc.2022.915833 (PMC9394455; doi:10.3389/fonc.2022.915833)
Supplement: Supplementary file 1 [file DataSheet_1.pdf]

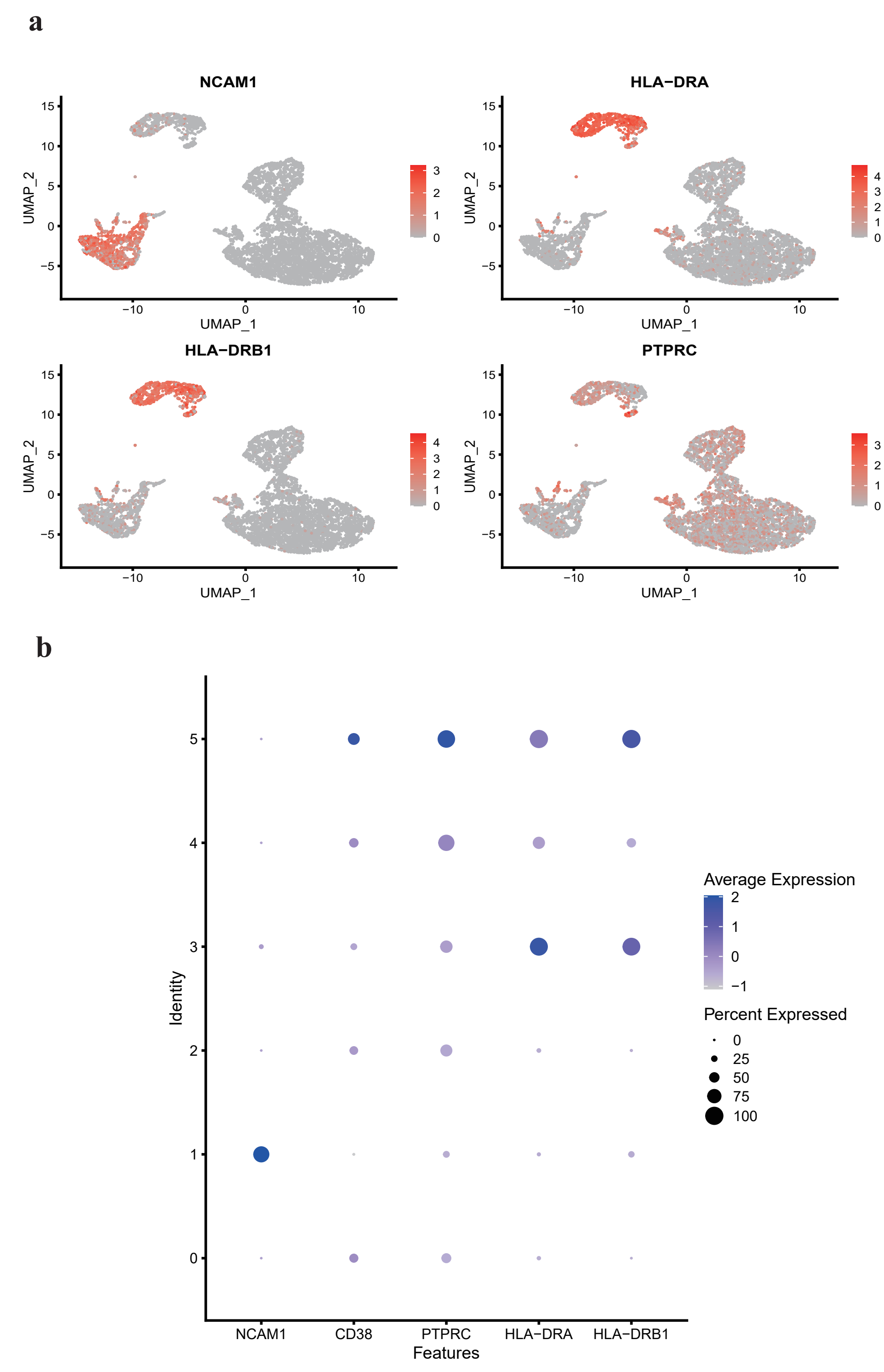

Fig.S1 The immunophenotype of *CBFA2T3-GLIS2* in AMKL

(A&B) The feature plots and dot plots of immunophenotype guess of *CBFA2T3-GLIS2*. *NCAM1*encodes CD56 and *PTPRC* encodes CD45.

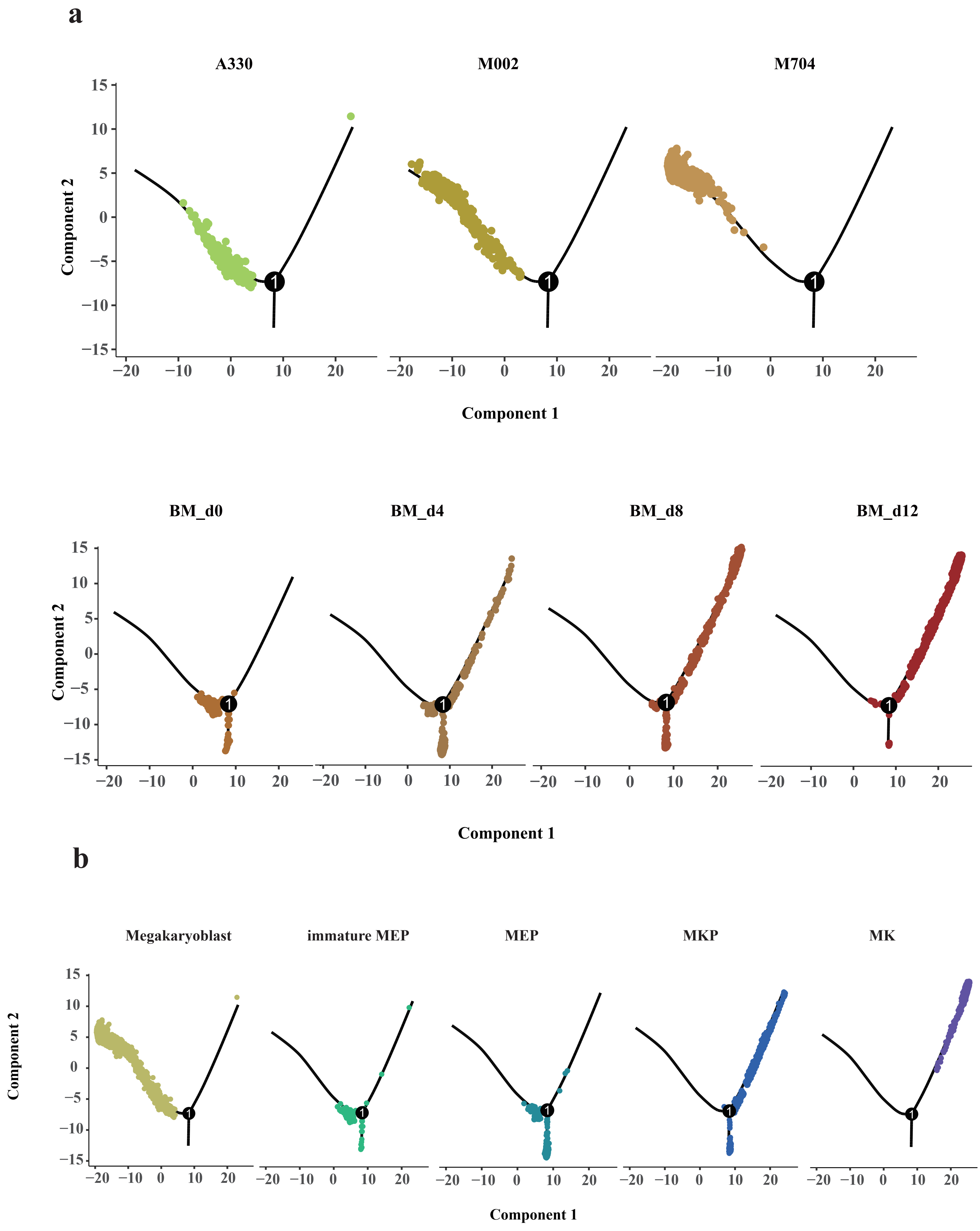

Fig.S2 The pseudo-time analysis of malignant cells in AMKL and MKs in hiBM combined.

(A) Pseudo-time analysis of abnormal and normal MK lineage cells. The time points of hiBM indicate the gradual maturity of MK lineage cells.(B) Pseudo-time analysis showing the abnormal megakaryoblasts and the MK lineage cells.

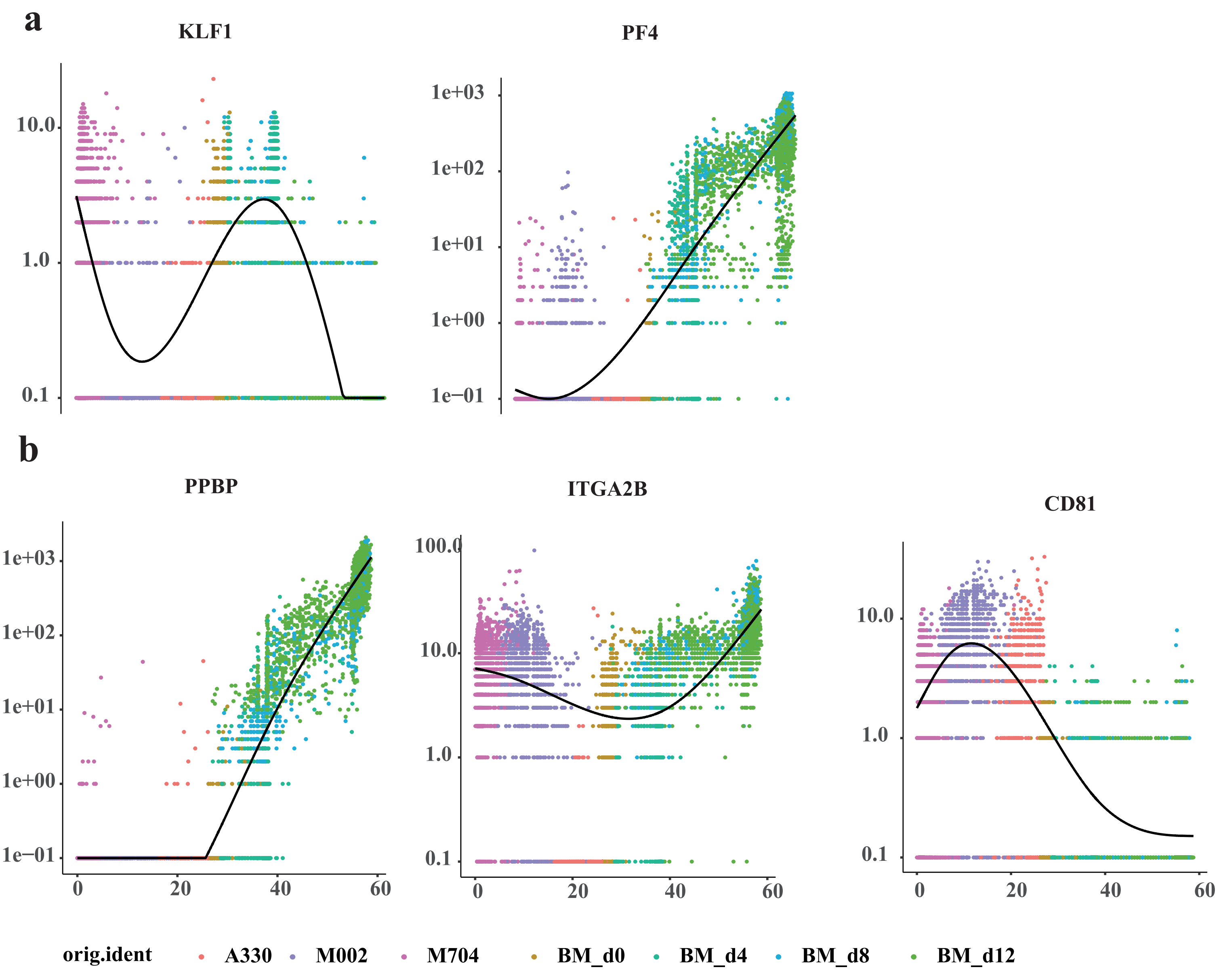

Fig.S3 Trajectory analysis of significant genes in AMKL cells and normal MKs.

(A) KLF1, the transcription factor (TF) of erythroid lineage; PF4 , the marker gene of MK. (B) PPBP, the signature gene of MK and platelet; ITGA2B, highly expressed in AMKL, MK, and platelet.
